# Transgenic A53T Mice Have Astrocytic α‐Synuclein Aggregates in Dopamine and Striatal Regions

**DOI:** 10.1111/nan.70097

**Published:** 2026-08-17

**Authors:** Cormac Peat, Asheeta Prasad, David I. Finkelstein, Caroline Sue, Jennifer A. Johnston, Clare L. Parish, Lachlan Thompson, Deniz Kirik, Glenda M. Halliday

**Affiliations:** ^1^ Charles Perkins Centre & Faculty of Medicine and Health School of Medical Sciences The University of Sydney Sydney New South Wales Australia; ^2^ Aligning Science Across Parkinson's (ASAP) Collaborative Research Network Chevy Chase Maryland USA; ^3^ Department of Optometry and Vision Sciences The University of Melbourne Parkville Victoria Australia; ^4^ Neuroscience Research Australia Sydney New South Wales Australia; ^5^ NysnoBio Mills Valley California USA; ^6^ The Florey Institute of Neuroscience and Mental Health & The University of Melbourne Parkville Victoria Australia; ^7^ BRAINS Unit, BMC D11 Lund University Lund Sweden; ^8^ Brain and Mind Centre & Faculty of Medicine and Health School of Medical Sciences The University of Sydney Sydney New South Wales Australia

**Keywords:** α‐synuclein, astrocyte, striatum, substantia nigra, ventral tegmental area

## Abstract

**Aims:**

The accumulation of α‐synuclein in astrocytes in Parkinson's disease is considered to be secondary to neuronal accumulation. The aim of this study to identify whether astrocytes accumulate small α‐synuclein aggregates before or after neurons in the nigrostriatal pathway.

**Methods:**

Fixed serial midbrain and striatal sections from M83 A53T transgenic mouse model of Parkinson's disease and wild‐type controls were histologically processed for multiplex labelling of α‐synuclein and astrocytic markers and astrocyte quantitation performed on digital images using QuPath software.

**Results:**

The density of astrocytes within the substantia nigra pars compacta was approximately 30% greater compared with other sampled regions (*p* < 0.005). Small aggregates of α‐synuclein were observed in astrocytic processes, including in wild‐type mice where a quarter of all astrocytes had an obvious α‐synuclein aggregate. Compared to wild‐type, A53T transgenic astrocytes had significantly enlarged somas (*p* < 0.001) with more processes (*p* < 0.001) consistent with a reactive phenotype. The A53T transgenic mice had more than double the numbers of astrocytes (*p* < 0.001) and 2.5 times more astrocytes with α‐synuclein aggregates compared to wild‐type mice (*p* < 0.001).

**Conclusions:**

These data suggest that small α‐synuclein aggregates are normally cleared by astrocytes and that the substantia nigra pars compacta requires more astrocytic support for this function than other midbrain dopaminergic regions or the striatum. This adds another vulnerability factor to those already known for the substantia nigra with early deficits in clearance of small α‐synuclein aggregates by astrocytes associated with increased astrocytic reactivity in the A53T transgenic mouse model.

## Summary

- The substantia nigra pars compacta contains a higher density of astrocytes than the ventral tegmental area or striatum, indicating a greater reliance on astrocytic function and a greater vulnerability to astrocyte dysfunction
- Small aggregates of α‐synuclein were observed in wild‐type midbrain and striatal astrocytes, indicating normal clearance of α‐synuclein by these astrocytes
- *SNCA* A53T transgenic mice have more than double the number of midbrain and striatal astrocytes and more reactive astrocytes containing α‐synuclein aggregates prior to α‐synuclein accumulation in neurons

## Graphical abstract

To identify whether astrocytes accumulate α‐synuclein aggregates before or after neurons, astrocytes from the midbrain and striatum of M83 A53T transgenic mice were compared to wild‐type. The substantia nigra pars compacta had more astrocytes than the ventral tegmental area or striatum, and small α‐synuclein aggregates were observed even within wild‐type astrocytes, indicating normal astrocytic clearance of α‐synuclein. A53T mice had more than double the number of astrocytes and more reactive astrocytes containing α‐synuclein aggregates prior to α‐synuclein accumulation in neurons.

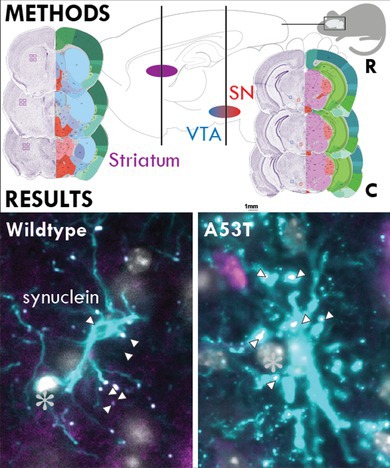

**Abbreviations**
ALDH1L1: 10‐formyltetrahydrofolate dehydrogenase
BSA: bovine serum albumin
CP: striatum
CYP27B1: 25‐hydroxyvitamin D 1‐α‐hydroxylase
GFAP: glial fibrillary acidic protein
PBS: phosphate buffered saline
PD: Parkinson's disease
ROI: region of interest
RRID: research resource identifier
S100β: S100 calcium binding protein B
SNC: substantia nigra pars compacta
TBS: tris‐buffered saline
VEGFA: vascular endothelial growth factor A
VTA: ventral tegmental area

## 1 Introduction

Parkinson's disease (PD) is a neurodegenerative disease diagnosed by a loss of dopamine neurons in the substantia nigra pars compacta (SNC) and the widespread accumulation of neuronal intracytoplasmic inclusions of α‐synuclein called Lewy pathologies [1]. Genetic mutations in or multiplications of the *SNCA* gene, which encodes α‐synuclein, cause familial PD, and the first mutation identified in *SNCA* was the A53T mutation [1]. Current concepts of the disease suggest renaming it biologically as neuronal α‐synuclein disease [2], but notably, not only do patients with PD deposit α‐synuclein in neuritic and neuronal Lewy inclusions, this neuronal pathology is accompanied by α‐synuclein deposition in astrocytes [3, 4, 5]. In fact, more astrocytes have α‐synuclein aggregates (40%–50%) than neurons (5%–25%) in regions with Lewy pathology in patients with PD [4, 6, 7]. The α‐synuclein depositing in astrocytes in PD is non‐amyloidogenic, truncated at both the N and C termini for degradation [8, 9] and in isolation (i.e., with limited coexisting neuropathologies) does not cause significant astrocytic reactivity, enlargement or proliferation [7, 10, 11, 12]. In fact, there is a negative correlation between increasing α‐synuclein accumulation and reduced astrocytic glial fibrillary acidic protein (GFAP) levels in the PD SNC suggestive of a suppressed reactive function [12, 13], a reduction in SNC protoplasmic astrocytes [13] and a unique expression of vitamin D‐activatin enzyme in PD astrocytes suggestive of an enhanced neuroprotective capability [14]. Understanding the astrocytic biology associated with α‐synuclein deposition in PD warrants further analysis, particularly when biologically renaming Lewy body diseases.

Few animal models recapitulate the diagnostic features of PD, but the homozygous A53T α‐synuclein transgenic line M83 is the most common model used [15, 16, 17]. This model expresses ~5× more human A53T α‐synuclein than mouse α‐synuclein and develops a delayed severe and complex motor impairment leading to paralysis and death, as well as age‐dependent intracytoplasmic neuronal α‐synuclein inclusions [15]. Of note, selective high expression (10× increase) of the same A53T transgene in mice astrocytes and not neurons or other brain cells initiates non‐cell autonomous killing of neurons in association with the accumulation of aggregated and truncated forms of α‐synuclein only in the astrocytes [18]. These mice develop an earlier‐onset, rapidly progressive paralysis due to a loss of SNC and spinal motor neurons, as well as a widespread, severe astrogliosis with increased GFAP and excitatory amino acid transporters causing blood–brain‐barrier dysfunction and excitotoxicity [18]. This shows that high levels of intracellular α‐synuclein activate astrocytes causing considerable dysfunction and surrounding neurodegeneration, something not seen in PD. Until recently, there had been no studies of astrocytic pathology in the most commonly used M83 mouse model of PD that expresses less of the A53T transgene in all brain cells and has a more slowly progressive and representative form of PD [15, 16, 17]. A recent study assessing the blood–brain‐barrier in this animal model with neuronal α‐synuclein accumulation at 8 months showed decreased expression of tight junction‐related proteins, along with increased vascular permeability and accumulation of oligomeric α‐synuclein in activated astrocytes in the brain [19], suggesting that intracellular α‐synuclein accumulation in astrocytes increases their reactivity and potential to become neurotoxic in these settings [18, 20]. This differs from the lack of astrocytic reactivity with increased α‐synuclein accumulation observed in the SNC of PD cases [7, 10, 11, 12].

There has been a lot of discussion on the concept and diversity of astrocyte reactivity [21] and this may play a role. Single cell transcriptomics has made it easier to assess astrocytic differences in both human and mice datasets [22]. These comparisons have identified that human astrocytes are much more susceptible to oxidative stress due to lower catalase and glucose‐6‐phosphate dehydrogenase levels [22]. They have also identified that astrocyte populations in the basal ganglia are similar in both humans and mice and that two subtypes are significantly reduced and two increased in PD [23]. Those sharply decreased in abundance in PD are astrocytes that ensheath neurons (OLIG2 + astrocyte subtype) [24] and reactive A1/inflammation astrocytes (see [25] for subtype), while those increased in abundance are reactive astrocytes involved in oxidase stress and the extracellular matrix (this type is associated with proteostasis in PD) as well as astrocytes restricted to the basal ganglia that are involved in cell–cell and cell‐extracellular matrix interactions (LUZP2 + astrocytes) [23]. These changes are similar to the decrease in protoplasmic and increase in fibrous types of astrocytes more recently identified in the PD SNC [13]. Single‐cell transcriptomics from the midbrain and striatum of transgenic A53T mice shows an increase in reactive astrocytes with upregulation of nuclear factor kappa‐light‐chain‐enhancer of activation B cells signalling and also vascular endothelial growth factor A (VEGFA) [26], consistent with the recent work on inflammation and blood–brain‐barrier disruption in this model [19]. There has been no histological assessment of the abundance of astrocyte types in the basal ganglia of M83 A53T transgenic mice and, while VEGFA+ astrocytes are intimately involved in cell–cell and cell‐extracellular matrix interactions in their role regulating the blood–brain‐barrier, whether a neuroprotective astrocyte subtype (containing vitamin D‐activating enzyme) [14] is increased/observed in this model of PD has not been assessed.

## 2 Materials and Methods

### 2.1 Animals

All animal procedures were conducted in agreement with the Australian National Health and Medical Research Council's published Code of Practice for the Use of Animals in Research, and approval granted by The Florey Institute of Neuroscience and Mental Health Animal Ethics committee (22–034‐FINMH). Brain tissue sections from 6‐month‐old M83 A53T transgenic mice (*n* = 5) and age‐matched wild‐type controls (*n* = 4) were provided by the Florey Institute of Neuroscience and Mental Health at University of Melbourne and used for analysis. Mice that carry the human A53T mutation driven by the mouse prion promoter (Jax Stock No: 004479; B6;C3‐Tg (Prnp‐SNCA*A53T)83Vle/J; A53T α‐synuclein transgenic line M83) were obtained from Jackson Laboratories (resource research identifier (RRID): IMSR_JAX:004479, Bar Harbor, Maine) and a stable breeding colony generated in‐house (genotype of all mice used in the study confirmed by qPCR) [27]. Mice were euthanised by saline perfusion with 80 mg/kg sodium pentobarbital before perfusion with 0.1‐M phosphate buffered saline (PBS). Brains were then collected, numbered and left overnight in 4% paraformaldehyde solution before transfer to a 30% sucrose/1‐M PBS solution, which was refreshed a minimum of two times. Coronal sections were then cut on a cryostat at −25°C and collected serially on 0.5% gelatinised Superfrost slides. Sections were stored desiccated at −80°C before the immunohistochemistry was performed.

For the midbrain, 30‐μm sections were sampled every 90 μm with three sections mounted per slide giving three sequential series of three sections for each midbrain available for experiments (see below).

For the striatum, 20‐μm sections were sampled every 200 μm with eight sections mounted per slide giving 10 sequential series of eight sections from each striatum available for experiments (see below).

### 2.2 Experimental Plan (Table 1)

**TABLE 1:** Experimental design.

Experiment	Purpose	Region analysed	# animals, slides, sections	Protein markers used
1	Identify astrocyte types and colocalisation of α‐synuclein	Striatum	2, 2, 16	GFAP, S100β, ALDH1L1
			2, 2, 16	GFAP, ALDH1L1, α‐synuclein
			2, 2, 16	S100β, ALDH1L1, α‐synuclein
			2, 2, 16	None
2	Assess targeted astrocyte markers associated with PD	Midbrain	6, 6, 18	GFAP, VEGFA, α‐synuclein
			6, 6, 18	GFAP, CYP27B1, α‐synuclein
		Striatum	6, 6, 48	GFAP, VEGFA, α‐synuclein
			6, 6, 48	GFAP, CYP27B1, α‐synuclein
			2, 2, 16	None
3	Quantify astrocytes containing α‐synuclein and their morphology	Midbrain	9, 9, 27	GFAP, S100β, ALDH1L1, α‐synuclein
		Striatum	9, 9, 27	GFAP, S100β, ALDH1L1, α‐synuclein

Abbreviations: ALDH1L1 = 10‐formyltetrahydrofolate dehydrogenase, CYP27B1 = 25‐hydroxyvitamin D 1‐α‐hydroxylase, GFAP = glial fibrillary acidic protein, S100β = S100 calcium binding protein B, VEGFA = vascular endothelial growth factor A.

Experiments were planned to (1) identify types of astrocytes (using multiple astrocyte markers) and assess whether any particular subtypes of astrocyte contained α‐synuclein protein, (2) assess targeted astrocyte markers identified previously as changed in PD (VEGFA [26] and vitamin D‐activating enzyme [14]) and (3) quantify the proportion of astrocytes containing α‐synuclein protein. Experiments were performed blinded to genotype.

### 2.3 Immunofluorescence

Briefly, sections were washed in 1X tris‐buffered saline (TBS) with 1.25% Triton X‐100 and blocked for 1 h in bovine serum albumin (BSA)/casein blocking solution (4% BSA (w/v), 1% casein (w/v), 1.5% glycine (w/v), 0.25% Triton X‐100 (v/v) in 1xPBS), then incubated with a combination of primary antibodies overnight at 4°C (see Tables 1 and 2). After washing with PBS, the tissue sections were incubated with appropriate fluorophore‐conjugated secondary antibodies for 3 h (see Table 2), then thoroughly washed with TBS, counterstained with Hoechst (Thermo Fischer Scientific Cat# 62249; 1 μg/mL) before a final wash and mounting with Prolong Diamond Antifade mounting medium (Invitrogen Cat# P36970), then coverslipped. Control experiments with incubation without primary antibodies gave no immunofluorescence signals.

**TABLE 2:** Primary and secondary antibodies used.

Marker	Primary antibodies	Secondary antibodies
GFAP	Chicken anti‐GFAP (Thermo Fisher Scientific Cat# PA1‐10004, RRID:AB_1074620; diluted 1:1000)	Goat anti‐chicken 488 (Thermo Fisher Scientific Cat# A‐11039, RRID:AB_2534096; diluted 1:250) or Goat anti‐chicken 594 (Thermo Fisher Scientific Cat# A‐11042, RRID:AB_2534099; diluted 1:250) or Goat anti‐chicken 647 (Thermo Fisher Scientific Cat# A‐21449, RRID:AB_2535866; diluted 1:250)
S100β	Goat anti‐S100β (R and D Systems Cat# AF1820, RRID:AB_2184423; diluted 1:1000)	Donkey anti‐goat 647 (Thermo Fisher Scientific Cat# A‐21447, RRID:AB_2535864; diluted 1:250) or Donkey anti‐goat 594 (Thermo Fisher Scientific Cat# A‐11058, RRID:AB_2534105; diluted 1:250)
ALDH1L1	Rabbit anti‐ALDH1L1 (Thermo Fisher Scientific Cat# PA5‐78622, RRID:AB_2735443; diluted 1:1000)	Goat anti‐rabbit 647 (Thermo Fisher Scientific Cat# A32733, RRID:AB_2633282; diluted 1:250) or Goat anti‐rabbit 594 (Thermo Fisher Scientific Cat# A‐11012, RRID:AB_2534079; diluted 1:250)
α‐Synuclein	Mouse anti‐alpha‐synuclein (BD Biosciences Cat# 610787, RRID:AB_398108; diluted 1:1000)	Goat anti‐mouse 488 (Thermo Fisher Scientific Cat# A‐11001, RRID:AB_2534069; diluted 1:250)
VEGFA	Rabbit anti‐VEGF (GeneTex Cat# GTX102643, RRID:AB_11174248; diluted 1000)	Goat anti‐rabbit 594 (Thermo Fisher Scientific Cat# A‐11012, RRID:AB_2534079; diluted 1:250)
CYP27B1	Rabbit anti‐CYP27B1 (Millipore Cat# ABN182, RRID:AB_2616605; diluted 1:1000)	Goat anti‐rabbit 594 (Thermo Fisher Scientific Cat# A‐11012, RRID:AB_2534079; diluted 1:250)

Abbreviations: ALDH1L1 = 10‐formyltetrahydrofolate dehydrogenase, CYP27B1 = 25‐hydroxyvitamin D 1‐α‐hydroxylase, GFAP = glial fibrillary acidic protein, S100β = S100 calcium binding protein B, VEGFA = vascular endothelial growth factor A.

For all experiments (Table 1), triple labelling immunofluorescence with non‐overlapping fluorophores (Table 2) was used to determine colocalisation. For experiment 3 (Table 1), GFAP, S100 calcium binding protein B (S100β) and aldehyde dehydrogenase 1 family member L1 (ALDH1L1) were all identified by the same fluorophore (Table 2) to give the maximum labelling of astrocytes for colocalisation with α‐synuclein and quantitation (nuclei were counterstained with Hoechst).

### 2.4 Image Capture, Processing and Analysis

Sections were visualised at 10× magnification with a Leica THUNDER 3D Imager and standardised regions 200 × 200 μm in size selected. Z stack images of these regions were captured at 1‐μm intervals for 10 μm at 40×/NA1.1 magnification (this z distance separated overlapping astrocytes), and an additional high magnification image (63×/NA1.4) taken to ensure resolution of intracellular astrocytic labelling and for morphological measurements. The brightness and contrast of the images was standardised. For the midbrain slide, one region in the SNC and one region in the ventral tegmental area (VTA) were captured at the three levels in the series (Figure 1). For the larger striatum, standardised rostral, mid and caudal levels were selected, and four regions captured per level in the series (Figure 1).

**FIGURE 1:**
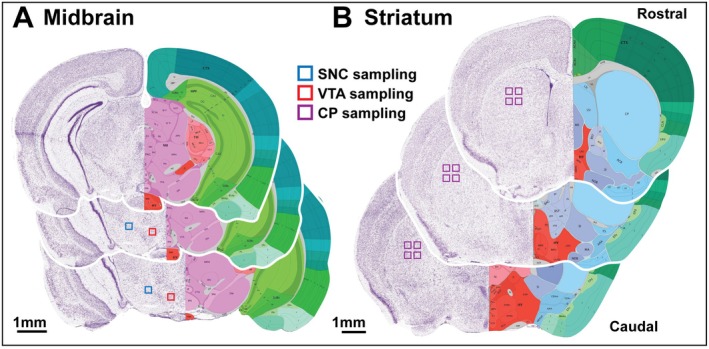
Sampling schematic for image capture and cell quantification in 200 × 200 μm sampling boxes. (A) For each of the three midbrain levels spaced 90 μm apart (anterior at top), a single box (blue) was sampled in the substantia nigra pars compacta (SNC) and another (red) in the ventral tegmental area (VTA) giving three sampling boxes per region. (B) For each of the three striatal (abbreviated CP) levels spaced 600 μm apart (anterior at top), four boxes (purple) were sampled at each level giving a total of 12 sampling boxes. Images are from standardised coronal Allen Adult Mouse Brain Atlas (https://mouse.brain‐map.org/experiment/thumbnails/100048576?image_type=atlas). All data are standardised to the sampling box area for each region of interest.

For experiment 3, 10–15 astrocytes displaying an obvious soma and clear processes were first established by separating the image by channel (AlexaFluor 594 for all astrocyte markers, experiment 3 in Table 1) in the high magnification images (63X/NA1.4) taken in each region of interest (ROI). These representative astrocytes were assessed for any changes in astrocyte morphology (soma size and the number of astrocyte processes). The soma of each astrocyte was traced using the polygon annotation tool in QuPath and the area measured. The number of processes for each astrocyte was also recorded.

In the entire ROI images, the widefield fluorescence Z stack images were exported as tiffs, processed and flattened using the QuPath software [28] for quantitation. The total number of astrocytes was established by separating the image by channel (AlexaFluor 594 for all astrocyte markers, experiment 2 in Table 1) and counting all astrocytes identified in the Z stack manually. Only astrocytes that displayed an obvious soma and clear processes were included. Once the total number of astrocytes was counted, double labelled astrocytes (astrocyte markers and α‐synuclein, see Table 1) were counted manually and the percentage of astrocytes expressing α‐synuclein determined by dividing the number of double‐labelled astrocytes by the total number of astrocytes.

Images were colour changed to ensure colour‐blind compatibility for presentation. Immunofluorescence for triple labelled GFAP + S100β + ALDH1L1 or for single labelled GFAP was changed to cyan, for single labelled S100β or α‐synuclein was changed to magenta, for single labelled ALDH1L1, VEGFA or for the vitamin D activator CYP27B1 was changed to yellow and for DAPI was changed to grey.

### 2.5 Statistical Analysis

Statistical analyses were performed using IBM SPSS Statistics (Version 29.0.2.0(20)), and data are presented as mean ± SEM. *p* < 0.05 was taken as statistically significant.

To assess the effects of genotype on regional astrocyte morphology and numbers, multivariate general linear models were used for the soma size of astrocytes and the number of astrocyte processes (genotype by region), as well as for the total astrocyte numbers and the proportion of astrocytes with α‐synuclein aggregates (genotype, region and level [see Figure 1]). Tukey post hoc tests were applied.

## 3 Results

### 3.1 Types of Astrocytes Observed in the Midbrain and Striatum

Astrocyte subtypes were analysed in the striatum where subtypes have been previously identified [23] as well as two regions of the midbrain. The different pan astrocyte markers used (Table 2) identified the same cells with similar morphologies, but the protein localisation for GFAP (astrocytic cytoskeletal marker), S100β (astrocytic calcium binding protein) and ALDH1L1 (astrocytic mitochondrial metabolising enzyme) within each astrocyte differed (Figure 2). In WT mice, GFAP was observed throughout the soma and processes of most astrocytes (less soma and more processes identified in controls, not shown), while ALDH1L1 and S100β had lower fluorescence within the processes of some astrocytes (Figure 2). Overall, the different markers did not label different astrocyte subtypes in the striatum or midbrain and therefore further experiments used either GFAP (which was observed in both the soma and processes of many astrocytes) or all three markers (to maximise the number of astrocytes observed) (Table 1).

**FIGURE 2:**
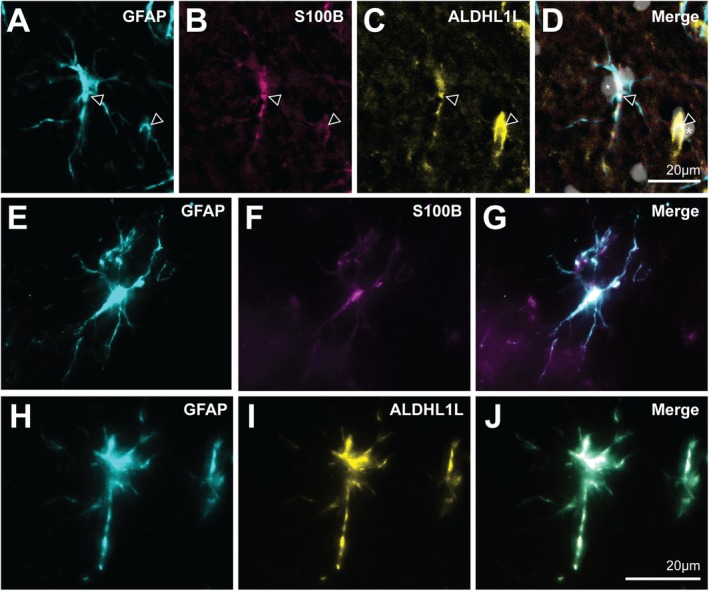
Evaluation of astrocyte markers for diverse astrocyte types. (A–D) Triple labelled immunofluorescence in the striatum of wild‐type mice using antibodies to the cytoskeletal protein GFAP (cyan in A), the calcium binding protein S100β (magenta in B) and the astrocytic mitochondrial metabolising enzyme ALDH1L1 (yellow in C). Scale in D is equivalent for A–C. These proteins were found co‐localised in the same astrocytes in the striatum (overlap white in D and arrowed, DAPI is grey and some nuclei asterisked). (E–G) Double labelled immunofluorescence of a striatal astrocyte showing GFAP (cyan in E) and S100β (magenta in F) co‐localisation (white in G). (H–J) Double labelled immunofluorescence of midbrain astrocytes showing GFAP (cyan in H) and ALDH1L1 (yellow in I) co‐localisation (white in J). Scale in J equivalent for E–J.

### 3.2 Most Astrocytes in A53T Transgenic Mice Appear Reactive

The morphology of the astrocytes in all regions examined in the A53T transgenic mice was consistent with reactive astrocytes where they upregulate core markers including the astrocytic cytoskeletal marker GFAP, the astrocytic calcium binding protein S100B and the astrocytic mitochondrial metabolising enzyme ALDH1L1 [21], all the markers used in the present study. Astrocytes in the A53T transgenic mice had significantly enlarged somas (Figures 3 and 4A–G, mean μm^2^ ± SEM A53T = 0.458 ± 0.035, WT = 0.216 ± 0.039, *F*(1) = 21, *p* < 0.001) across all regions (*F*(2) = 0.32, *p* = 0.728) and an increased number of processes (Figures 3 and 4A–F,H, mean ± SEM A53T = 4.19 ± 0.14, WT = 3.10 ± 0.16, *F*(1) = 27, *p* < 0.001) across all regions (*F*(2) = 0.17, *p* = 0.849) compared with WT mice.

**FIGURE 3:**
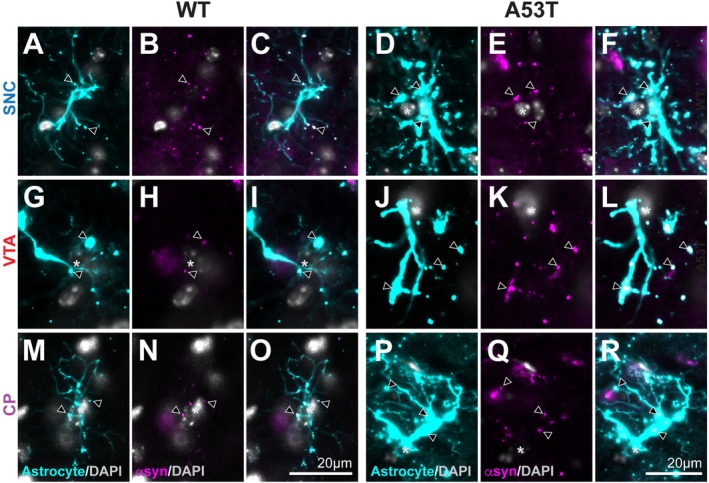
α‐Synuclein puncta are common in astrocytic processes. Immunofluorescence labelling of astrocytes using all three astrocytic proteins (GFAP, S100β and ALDH1L1 cyan) and α‐synuclein (magenta) in the substantia nigra pars compacta (SNC, A–F), ventral tegmental area (VTA, G–L) and striatum (CP, M–R) of wild‐type (WT, A–C, G–I, M–O) and A53T transgenic (A53T, D–F, J–L, P–R) mice showing α‐synuclein aggregates in their processes (white arrowheads). Grey = nuclear DAPI. Scale bar in O is equivalent for WT micrographs (A–C, G–I, M–O). Scale bar in R is equivalent for A53T micrographs (D–F, J–L, P–R).

**FIGURE 4:**
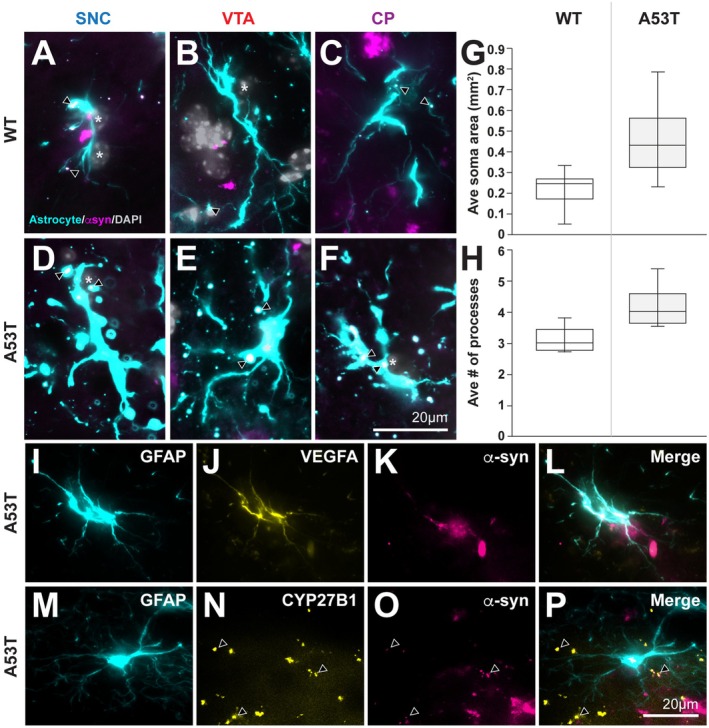
Evidence of reactive astrocytes in A53T transgenic mice. (A–F) Immunofluorescence labelling of astrocytes using all three astrocytic proteins (GFAP, S100β and ALDH1L1 cyan) and α‐synuclein (magenta) in the substantia nigra pars compacta (SNC, A, D), ventral tegmental area (VTA, B, E) and striatum (CP, C, F) of wild‐type (WT, A–C) and A53T transgenic (A53T, D–F) mice showing α‐synuclein aggregates in their processes (white arrowheads). Grey = nuclear DAPI. Scale bar in F is equivalent for A–E. (G) Boxplot of the median astrocyte soma area and the interquartile range (in μm^2^) in wild‐type (WT, *n* = 4) and A53T transgenic (A53T, *n* = 5) mice showing more than a doubling in the soma area of astrocytes in A53T versus WT mice across all regions assessed using multivariate linear regression (*F*_soma size_(1) = 21, *p* < 0.001; *F*_region_(2) = 0.32, *p* = 0.728) consistent with a reactive phenotype. (H) Boxplot of the median number of processes per astrocyte and the interquartile range in wild‐type (WT, *n* = 4) and A53T transgenic (A53T, *n* = 5) mice showing a 35% increase in the number of astrocytic processes in A53T versus WT mice across all regions assessed using multivariate linear regression (*F*_processes_(1) = 27, *p* < 0.001; *F*_region_(2) = 0.17, *p* = 0.849) consistent with a reactive phenotype. (I–L) Triple labelled immunofluorescence in the striatum of A53T transgenic (A53T) mice using antibodies to the cytoskeletal protein GFAP (cyan in I), the growth factor for vasculature and blood–brain‐barrier VEGFA (yellow in J) and α‐synuclein (magenta in K) showing that reactive astrocytes in A53T mice with α‐synuclein aggregates express VEGFA (white in L) consistent with increased reactivity as recently identified [19]. (M–P) Triple labelled immunofluorescence in the striatum of A53T transgenic (A53T) mice using antibodies to the cytoskeletal protein GFAP (cyan in I), the synthesising enzyme for vitamin D CYP27B1 (yellow in J) recently identified throughout the cytoplasm of astrocytes that sequester α‐synuclein in sporadic Parkinson's disease [14], and α‐synuclein (magenta in K) showing that reactive astrocytes in A53T mice with α‐synuclein aggregates do not express CYP27B1 throughout their cytoplasm, but can have rare vesicular‐like aggregates (white arrowheads).

Assessment of example functional markers also indicated that the reactive astrocytes in A53T transgenic mice had functional changes. VEGFA has been linked to vascular coupling and blood–brain‐barrier integrity and was assessed to see if most reactive astrocytes in the A53T transgenic mice were also VEGFA+ (Figure 4I–K), as recently published [19]. We confirm this finding. In contrast to the specific expression of the synthesising enzyme for vitamin D CYP27B1 in the entire cytoplasm in astrocytes that sequester α‐synuclein in the brains of sporadic PD patients [14], this PD‐specific change was not observed in A53T transgenic mice (Figure 4M–P). Only small vesicles containing both CYP27B1 and α‐synuclein were found in A53T transgenic mice astrocytes with α‐synuclein aggregates (Figure 4M–P).

### 3.3 Most Astrocytes in A53T Transgenic Mice Accumulate α‐Synuclein

Assessment of astrocytes combining all astrocyte marker proteins together allowed their full morphology to be identified (Figures 3 and 4A–F) and assessment of α‐synuclein aggregates within them easier to identify. Small aggregates of α‐synuclein were observed mostly in astrocytic processes, even in WT mice (Figures 3 and 4A, arrowheads) where just over one in four astrocytes had an obvious α‐synuclein aggregate in one or more processes (Figure 5B). This suggests that aggregates of α‐synuclein are normally cleared by astrocytes.

**FIGURE 5:**
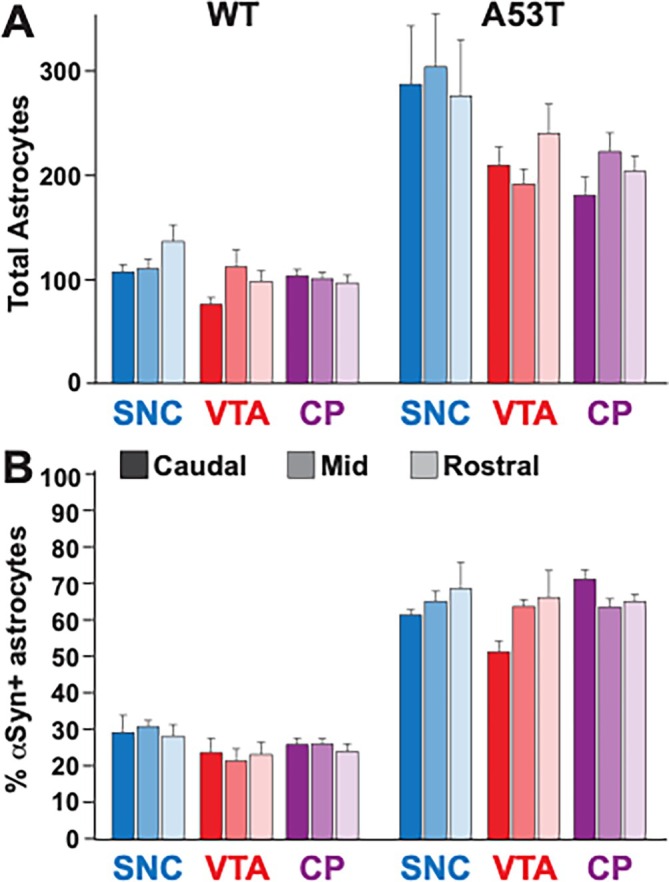
Evidence for a significant increase in the number of astrocytes in A53T transgenic mice. (A) Graph of the total number of astrocytes per regional sample box by genotype (wild‐type WT *n* = 4 and A53T transgenic *n* = 5), region (substantia nigra pars compacta SNC, ventral tegmental area VTA and striatum CP) and regional level (rostral, middle and caudal, see Figure 1) showing no significant differences between the density of astrocytes at different levels of the same regions using multivariate linear regression (*F*_location_(2) = 0.33, *p* = 0.72). Multivariate linear regression revealed that the SNC had an increased density of astrocytes (approximately 30% more) compared with the VTA and CP (*F*_region_(2) = 6.8, *p* = 0.002, posthoc tests between SNC and other regions *p* < 0.005) and that A53T mice had more than double the average number of astrocytes per sample box compared with WT mice (*F*_genotype_(1) = 104, *p* < 0.001) with no genotype by region interactions (*F*(2) = 3.085, *p* = 0.053). (B) Graph of the proportion of astrocytes containing α‐synuclein aggregates by genotype (wild‐type WT *n* = 4 and A53T transgenic *n* = 5), region (substantia nigra pars compacta SNC, ventral tegmental area VTA and striatum CP) and regional level (rostral, middle and caudal, see Figure 1) showing no significant differences between proportion of α‐synuclein‐containing astrocytes at different levels of the same regions using multivariate linear regression (*F*(2) = 0.077, *p* = 0.93). Multivariate linear regression revealed that the VTA had less α‐synuclein‐containing astrocytes (just over 10% less) compared with the SNC (*F*_region_(2) = 3.158, *p* = 0.049, posthoc tests between SNC and VTA *p* < 0.02) and that A53T transgenic mice had nearly a 2.5× greater proportion of astrocytes with α‐synuclein aggregates compared to WT mice (*F*_genotype_(1) = 494, *p* < 0.001) with no genotype by region interactions (*F*(2) = 0.905, *p* = 0.41).

Multivariate analysis revealed a significant difference in the density of astrocytes and the proportion of astrocytes containing α‐synuclein aggregates (Figure 5) between genotype (*F*(2) = 420, *p* < 0.001) and region (*F*(4) = 5.95, *p* < 0.001) but not level within region (*F*(4) = 0.291, *p* = 0.88). There was no genotype by region interactions (*F*(2, 4) = 1.54, *p* = 0.20). In A53T transgenic mice, the majority of astrocytes contained α‐synuclein aggregates (Figure 5B), and compared with WT astrocytes, the α‐synuclein aggregates were observed in more than one process and often also within their soma (Figures 3 and 4D–F). Diffuse α‐synuclein accumulations in occasional neurons was also seen in each of the areas analysed (data not shown), although frank Lewy pathology was not observed. The A53T transgenic mice had more than double the average number of astrocytes per sample box compared with WT mice (mean ± SEM A53T = 249 ± 10, WT = 104 ± 11, *F*(1) = 104, *p* < 0.001, Figure 5A). A53T transgenic mice also had nearly a 2.5× greater proportion of astrocytes with α‐synuclein aggregates compared to WT mice (mean ± SEM A53T = 64 ± 1%, WT = 26 ± 1%, *F*(1) = 494, *p* < 0.001, Figure 5B). There were approximately 30% more astrocytes in the SNC compared with the VTA and striatum (CP) (mean ± SEM SNC = 213 ± 12, VTA = 162 ± 12, CP = 153 ± 12, *F*(2) = 6.8, *p* = 0.002, post hoc *p* < 0.005, Figure 5A) suggesting this region requires more astrocytic support.

## 4 Discussion

PD is considered to be a neuronal α‐synuclein disease [2], but the main neuronal pathologies are accompanied by significantly more α‐synuclein deposition in astrocytes, which is poorly understood [3, 4, 5, 7]. The astrocytes with α‐synuclein accumulation in sporadic PD do not have a reactive phenotype [7, 10, 11, 12] (unless Alzheimer co‐pathology occurs) [29, 30] and have neuroprotective properties, like expressing the vitamin D‐activating enzyme particularly around neurons without Lewy pathologies [14]. Cell studies have identified that astrocytes can ingest enormous amounts of α‐synuclein of different forms and are activated to degrade the α‐synuclein [31, 32, 33, 34]. In these models, astrocytes can store α‐synuclein due to incomplete lysosomal degradation, which with progressive accumulation can cause mitochondrial impairments in the astrocytes and senescence, affecting their function [32, 35]. This is likely to be relevant for PD as this important astrocyte function can be induced by PD‐linked environmental factors [36]. These studies suggest that effective astrocyte function is essential for adequate removal of pathogenic α‐synuclein and that factors impacting on this function increase α‐synuclein accumulation in the brain.

The normal accumulation of the prevalent protein α‐synuclein in astrocytes has not been identified in the brains of WT mice previously, but the careful examination of astrocytes in the present study revealed α‐synuclein accumulations in the processes of astrocytes (Figures 3 and 4A,C). Just over one in four WT astrocytes had α‐synuclein aggregates in their processes (Figure 5B). Of note, no α‐synuclein accumulations were observed in WT neurons in these regions, suggesting that astrocytes normally remove any excess α‐synuclein. This has only been observed previously in in vitro experiments where similar isolated α‐synuclein puncta are found in around 40% of astrocytes after 24 h [32] with phagocytic accumulation of α‐synuclein peaking at 48 h and the α‐synuclein degraded by 170 h [31]. Interestingly, the SNC has approximately 30% more astrocytes than the VTA or CP in mice (Figure 5A) suggesting that more astrocyte support is required in this region. In the SNC the density of neurons is significantly increased as the name suggests (compact) and in humans a single astrocyte is associated with each dopamine neuron in the SNC whereas in the CP a single astrocyte is associated with six to eight neurons [37]. The increased astrocytes in the SNC in mice could indicate a similar working arrangement in the SNC of mice where a single astrocyte cares for each dopamine neuron. Together these data confirm the importance of astrocytes in disposing of excess α‐synuclein in normal healthy brain tissue and that this relationship appears most intimate in the SNC. While speculative, this process may be related to astrocyte‐dependent circuit remodelling by synapse phagocytosis that is essential for synaptic homeostasis [38]. This is a process that has not been well studied in these regions in mice but astrocytic synaptic and dendritic spine remodelling are known to occur in progressive models of PD [39, 40].

Animal models of PD have not assessed astrocytic accumulation of α‐synuclein to any extent. In the present study assessment of astrocytes was performed at 6 months prior to the onset of motor dysfunction and dopamine neuron loss, which begin between 8 and 12 months of age in this model. Accumulation of oligomeric α‐synuclein in activated astrocytes in the brain of 8‐month‐old A53T transgenic mice was reported recently in association with decreased expression of tight junction‐related proteins and increased vascular permeability [19]. As this study focussed on blood–brain‐barrier disruption in the symptomatic stage, no general quantitation of the impact of any α‐synuclein accumulation on astrocytes was evaluated. The present study quantitatively assessed the reactivity of astrocytes prior to symptom onset and found significant accumulation of α‐synuclein in association with increased astrocyte reactivity in A53T transgenic mice at 6 months. The astrocytes in this common PD model did not have dispersed α‐synuclein accumulation throughout their cytoplasm at this age, as often seen in end‐stage PD in areas with significant Lewy pathology [3, 4, 5, 7], but they had an increase in the presence of α‐synuclein aggregates in their processes as well as in their somas (Figures 3 and 4D–F). In fact, most astrocytes contained α‐synuclein aggregates in this PD mouse model at this early stage (Figure 5B). While the astrocytes in the A53T transgenic mice were more reactive (twice as many that were double the size with more processes) (Figures 4G,H and 5A), their increased size appeared commensurate with the increase in the amount of α‐synuclein aggregates in each astrocyte, suggestive of an increase in the turnover of these aggregates by these more reactive astrocytes. Similar morphological changes in perisynaptic astrocytes have been described in the rat CP following 6‐OHDA dopaminergic denervation [41, 42]. At the age assessed (6 months), the A53T transgenic mice had diffuse α‐synuclein accumulations in some neurons in each of the areas analysed (data not shown), although frank Lewy pathology was not observed. This indicates that the increased astrocytic α‐synuclein occurs before significant neuronal α‐synuclein begins in this model. This is also observed in the preformed fibril mouse model with injection into the CP where astrocyte reactivity occurs significantly earlier than α‐synuclein inclusion formation in SNC dopaminergic neurons and is associated with striatal dopamine terminal denervation [43].

As recently detailed at 8 months [19], the reactive astrocytes in the A53T transgenic mice also contained VEGFA at 6 months (before symptom onset), an indicator of functional upregulation of vascular coupling, increased hypoxia sensing and reduced blood–brain‐barrier integrity. Reduced blood–brain‐barrier integrity and an increase in striatal blood vessel density have been shown in human α‐synuclein overexpressing mice at 8 months, followed at 13 months (now symptomatic) by more significant accumulation of α‐synuclein, a decrease in dopamine terminals and a decrease in blood vessel density [44]. These data suggest that blood–brain‐barrier disruption occurs with increasing astrocytic VEGFA and α‐synuclein accumulation in astrocytes. While there is limited data on VEGFA in the PD brain, elevated immunoreactivities in the SNC but not striatal astrocytes with increased GFAP have been reported [19, 45] with an increase in vascular endothelial cells [46] suggesting some neovascularisation. In the adult rat rotenone model of PD, the SNC also has an increase in the number of astrocytes and blood capillaries due to an increase in string vessels [47]. SNC string vessel formation has been identified in PD brain with increased astrocytes in the striatum of the same cases reducing this effect in this location [48]. The data suggest that astrocyte function supporting blood vessels in the SNC in particular is disrupted with increasing astrocytic α‐synuclein accumulation.

As indicated, the highly reactive astrocytes observed in the PD model studied do not occur in the PD SNC [7, 10, 11, 12], which may be a function of the disease stage studied (early in the present study, later in most human tissue studies) or could be due to the expression of A53T human α‐synuclein in astrocytes in this model. If the latter, the morphology and pattern of α‐synuclein expression are extremely similar to that observed in WT astrocytes which are known to have considerably reduced α‐synuclein expression compared to neurons [49]. This could suggest increased α‐synuclein uptake although intracellular expression cannot be ruled out. In humans with sporadic PD there is a neuroprotective upregulation of the synthesising enzyme for vitamin D CYP27B1 throughout the cytoplasm of the 50% of astrocytes that contain α‐synuclein puncta and are associated with neurons that do not contain Lewy pathology [14]. However, the mice astrocytes in the present study did not have widespread astrocytic cytoplasmic CYP27B1 that indicates a neuroprotective phenotype, but did have restricted co‐localisation of CYP27B1 and α‐synuclein within astrocytic puncta (Figure 4M–P). This may suggest the initiation of neuroprotection against the incomplete lysosomal degradation of α‐synuclein in the astrocytes at this more early stage of synucleinopathy. In the rat rotenone model, vitamin D treatment before and after the lesion partially restores SNC and striatal tyrosine hydroxylase expression [50, 51], and in three out of four clinical trials of vitamin D supplementation in PD, enhanced physical performance and reduced L‐dopa dyskinesias were reported [52], suggesting that vitamin D can ameliorate cellular dysfunctions associated with α‐synuclein aggregations. This function may be initiated in astrocytes at a later disease stage to that assessed in the present study.

Overall, we have shown that astrocytes in this A53T transgenic mouse model upregulate their clearance of mutant α‐synuclein aggregates from the tissue analysed, suggesting this upregulation occurs earlier than any substantial neuronal accumulation. As indicated, the tissue changes are most consistent with astrocyte‐dependent circuit remodelling by synapse phagocytosis due to similar α‐synuclein accumulations observed in WT mice. While the CP terminals most amenable to synaptic remodelling are likely to be dopaminergic terminals, the afferents to the SNC that might be involved could be either from the CP, prefrontal, cingulate, insular and/or somatomotor cortices [53] or from the retrorubral field that contains the A8 dopaminergic cell group [54]. The loss of nigral dopamine release [55] or the selective loss of A8 dopamine neurons [56] induces progressive Parkinsonism in animal models, suggestive that the terminals most targeted for remodelling could be the dopaminergic terminals in all regions. Importantly, in early PD there is a loss of presynaptic terminals only in the SNC that accompanies the loss of dopamine function in synapses in the CP at the same time [57], a finding recently validated and shown to increase with disease progression [58]. While speculative, a loss of the ability of astrocytes to remodel synapses and mop up α‐synuclein in these regions may be the precipitating event for the degeneration in PD.

## Author Contributions

Conceptualisation and study design: Cormac Peat, Asheeta Prasad and Glenda Halliday as part of a larger funded project designed by Carolyn Sue, Jennifer Johnston, Claire Parish, Lachlan Thompson, Deniz Kirik and Glenda Halliday. Reagent/materials/analysis tools: David Finkelstein, Asheeta Prasad and Glenda Halliday. Data acquisition: Cormac Peat. Data analysis and interpretation: Cormac Peat, Asheeta Prasad and Glenda Halliday. Drafting of the manuscript: Cormac Peat and Glenda Halliday. Editing of the manuscript: All authors. All authors have read and approved the final manuscript.

## Funding

This work was supported in part by Aligning Science Across Parkinson's (000497) through the Michael J. Fox Foundation for Parkinson's Research (MJFF) and also by funding from the National Health and Medical Research Council of Australia to G.M.H. (Investigator Grant 1176607) and C.L.P. (Investigator Grant 2026395). For open access, the authors have applied a CC BY public copyright licence to all Author Accepted Manuscripts arising from this submission.

## Ethics Statement

All animal procedures were conducted in agreement with the Australian National Health and Medical Research Council's published Code of Practice for the Use of Animals in Research, and approval granted by The Florey Institute of Neuroscience and Mental Health Animal Ethics committee (22‐034‐FINMH).

## Conflicts of Interest

The authors declare no conflicts of interest.

## Data Availability

The data, code, protocols and key lab materials used and generated in this study are listed in a Key Resource Table alongside their persistent identifiers at Zenodo https://zenodo.org/records/18345432. Images analysed are deposited at https://www.ebi.ac.uk/biostudies/studies/S‐BIAD2485 and generated data and readme files are deposited at Zenodo, https://zenodo.org/records/18345432.
